# Relative biological effectiveness of oxygen ion beams in the rat spinal cord: Dependence on linear energy transfer and dose and comparison with model predictions

**DOI:** 10.1016/j.phro.2024.100581

**Published:** 2024-04-20

**Authors:** Christin Glowa, Maria Saager, Lisa Hintz, Rosemarie Euler-Lange, Peter Peschke, Stephan Brons, Michael Scholz, Stewart Mein, Andrea Mairani, Christian P. Karger

**Affiliations:** a Department of Medical Physics in Radiation Oncology, German Cancer Research Center (DKFZ), Heidelberg, Germany; b National Center for Radiation Research in Oncology (NCRO), Heidelberg Institute for Radiation Oncology (HIRO), Heidelberg, Germany; c Department of Radiation Oncology and Radiotherapy, University Hospital Heidelberg, Germany; d Faculty of Biosciences, Heidelberg University, Heidelberg, Germany; e Department of Radiooncology/Radiobiology, German Cancer Research Center (DKFZ), Heidelberg, Germany; f Heidelberg Ion Beam Therapy Center (HIT), Heidelberg, Germany; g Department of Biophysics, Helmholtz Center for Heavy Ion Research (GSI), Darmstadt, Germany; h Clinical Cooperation Unit Translational Radiation Oncology, German Cancer Research Center (DKFZ), Heidelberg, Germany; i National Centre of Oncological Hadrontherapy (CNAO), Medical Physics, Pavia, Italy

**Keywords:** Oxygen ion radiotherapy, Relative biological effectiveness (RBE), Linear energy transfer (LET), Rat spinal cord, Myelopathy, Late normal tissue effects, Local effect model (LEM), Modified microdosimetric kinetic model (mMKM)

## Abstract

**Background and purpose:**

Ion beams exhibit an increased relative biological effectiveness (RBE) with respect to photons. This study determined the RBE of oxygen ion beams as a function of linear energy transfer (LET) and dose in the rat spinal cord.

**Materials and methods:**

The spinal cord of rats was irradiated at four different positions of a 6 cm spread-out Bragg-peak (LET: 26, 66, 98 and 141 keV/µm) using increasing levels of single and split oxygen ion doses. Dose-response curves were established for the endpoint paresis grade II and based on ED_50_ (dose at 50 % effect probability), the RBE was determined and compared to model predictions.

**Results:**

When LET increased from 26 to 98 keV/µm, ED_50_ decreased from 17.2 ± 0.3 Gy to 13.5 ± 0.4 Gy for single and from 21.7 ± 0.4 Gy to 15.5 ± 0.5 Gy for split doses, however, at 141 keV/µm, ED_50_ rose again to 15.8 ± 0.4 Gy and 17.2 ± 0.4 Gy, respectively. As a result, the RBE increased from 1.43 ± 0.05 to 1.82 ± 0.08 (single dose) and from 1.58 ± 0.04 to 2.21 ± 0.08 (split dose), respectively, before declining again to 1.56 ± 0.06 for single and 1.99 ± 0.06 for split doses at the highest LET. Deviations from RBE-predictions were model-dependent.

**Conclusion:**

This study established first RBE data for the late reacting central nervous system after single and split doses of oxygen ions. The data was used to validate the RBE-dependence on LET and dose of three RBE-models. This study extends the existing data base for protons, helium and carbon ions and provides important information for future patient treatments with oxygen ions.

## Highlights

• Oxygen ions were significantly more effective in the rat spinal cord than photons.
• The effectiveness of oxygen ions rose with linear energy transfer up to 98 keV/µm.
• At 141 keV/µm, the effectiveness of oxygen ions was decreased.
• Oxygen ions were more effective for split than for single doses.
• Prediction accuracy of the effectiveness of oxygen ions was model-dependent.

## 1 Introduction

Carbon ion radiotherapy presents a well-established modality for the treatment of critically localized, radioresistant tumors [1], [2], [3], [4]. While the favorable depth dose profile (spread-out Bragg-peak, SOBP) is comparable to that of protons, the increased relative biological effectiveness (RBE) caused by the higher linear energy transfer (LET) is expected to improve clinical outcome [5]. The RBE is defined as the ratio of *iso*-effective photon and ion doses and is predicted by mathematical models [6], [7]. Up to now, the mixed-beam model (MBM) [8], the local effect model (LEM) [9] and the modified microdosimetric kinetic model (mMKM) [10] have been applied for carbon ions in patients.

Carbon ions were selected based on the assumption that they exhibit a high peak-to-plateau RBE ratio together with a decreased oxygen enhancement ratio (OER) [11], which would especially be beneficial for the treatment of hypoxic tumors. However, the optimal ion type is not yet determined. While preclinical tumor studies showed that the peak-to-plateau RBE ratio of helium ions is limited and that it is even below one for argon ions [12], [13], carbon and neon ions exhibited higher ratios [13], [14] and a decreased OER in the SOBP [15], [16]. However, the clinical potential of ion beams depends also critically on the RBE in the late reacting normal tissue, e.g. the central nervous system (CNS). A number of studies have been performed in the rat spinal cord positioned in the entrance and SOBP regions [17], [18], [19], [20], [21], [22]. While these studies initially focused on helium ions, they were extended to carbon and neon ion beams employing either single, 4 or 8 (only neon) fractions (Fx) [22]. A further study has been performed for carbon ions with the rat spinal cord in the entrance region [23].

Although these studies provided important information on the RBE of passive ion beams in the CNS [24], they were not designed to benchmark RBE-models [9], [10], [25] developed more recently for active pencil beam scanning [26]. To validate the LEM for carbon ions, dedicated studies with, 1, 2, 6 and 18 fractions were performed with the rat spinal cord being positioned in the entrance region of a monoenergetic beam or at the center of a 1 cm SOBP [27], [28]. For a more detailed evaluation of the predicted RBE as a function of LET and dose, additional experiments with 1, 2 and 6 fractions were executed with the rat spinal cord positioned at six positions within a 6 cm SOBP [29], [30], [31], [32].

After extending these experiments to protons and helium ions [33], [34], this study measures the RBE of single and split oxygen ion doses in the rat spinal cord using the same setup as in previous studies. The obtained RBE values are compared with those of carbon ions and are used to benchmark predictions of different RBE-models.

## 2 Materials and methods

### 2.1 Animals

In total, 280 young adult female Sprague-Dawley rats (Charles River, Sulzfeld, Germany) at age of 10 ± 2 weeks (mean ± range) and 207 ± 15 g (mean ± SD) weight were used for the dose–response experiments, including 10 unirradiated controls. A gaseous mixture of 4 % Sevoflurane (Abbott, Wiesbaden, Germany) and oxygen at 2 l/min was used to keep animals under anesthesia during irradiation. Rats were housed under standard conditions at the Center for Preclinical Research of the German Cancer Research Center and all experiments were approved by the governmental review committee on animal care (ref. no. 35–9185.81/G-39/15).

### 2.2 Experimental setup

**Fig. 1:**
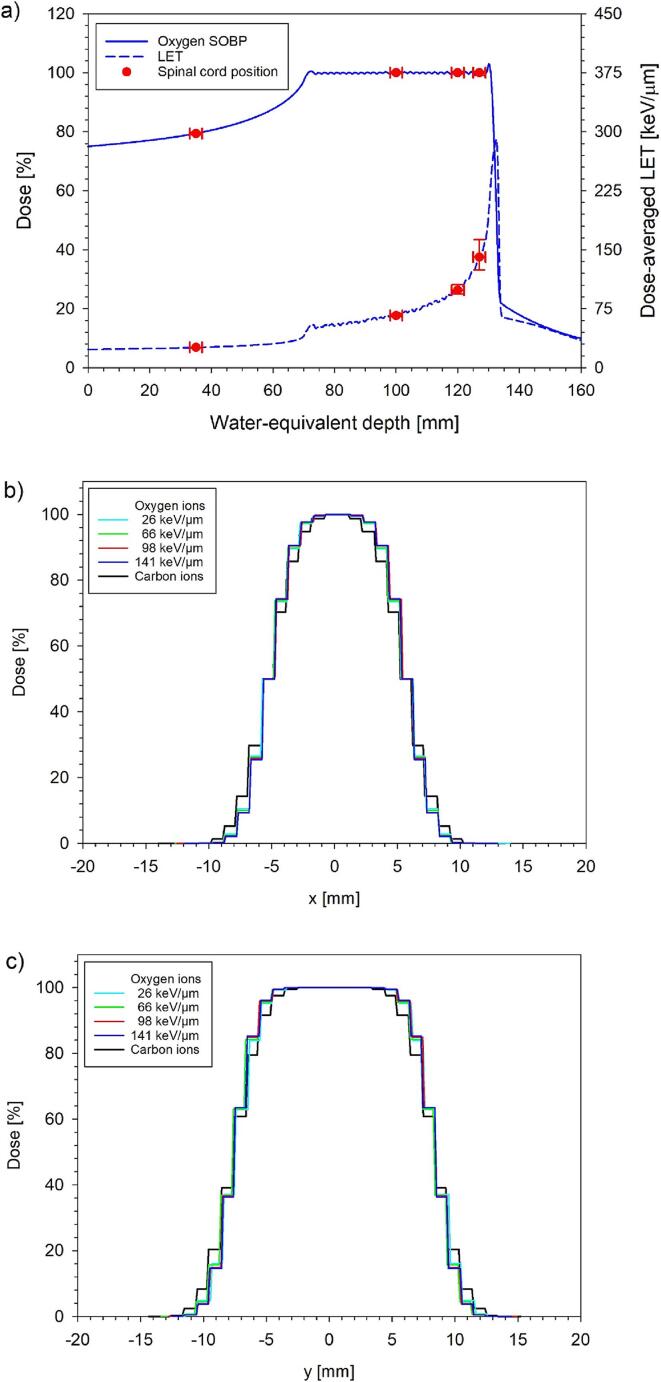
Dose- and LET-profiles along the oxygen ion SOBP (a) and the corresponding transversal dose profiles in comparison to those of carbon ions (b, c) used for the rat spinal cord irradiations. The dots mark the positions of the spinal cord. Horizontal and vertical error bars indicate the assumed 2-mm range uncertainty and the corresponding dose and LET uncertainty.

The experimental setup for the oxygen ion irradiations of the rat spinal cord was the same as for previous experiments with carbon ions [27], [28], [29], [30], [31], [32] (for details, see [27]). The cervical rat spinal cord (segments C1-C6) was irradiated from the ventral direction using a horizontal beam with the animals fixed in a hanging position [27], [29]. Polymethyl-methacrylate (PMMA)-boli were used to adjust the position of the spinal cord to four different positions (35, 100, 120 and 127 mm) within a 6 cm SOBP (70 to 130 mm water-equivalent depth, energy range 220–310 MeV/u) corresponding to dose-averaged LET-values of 26, 66, 98 and 141 keV/µm (Fig. 1a), respectively. For each of the four positions, irradiations were performed with either single or two equal (split) oxygen ion doses separated by 24 h. Increasing dose levels with 5 animals per dose group were used to measure dose–response curves covering 0–100 % effect probability. Table 1 displays the number of animals and the dose levels employed at each depth/LET for the single and split dose experiments.

**Table 1:** Total absorbed doses and number of animals used for the dose–response curves. At each dose level 5 animals were irradiated. 10 unirradiated animals served as controls.

**Spinal cord position within SOBP [mm]**	**LET [keV/µm]**	**Dose levels [Gy]**	**Total number of animals**
*Single dose*			
35	26	15.5, 16.5, 17.5, 18.5, 19.5, 20.5	30
100	66	12.5, 13.5#, 14.5, 15.5, 16.5#, 17.5	30
120	98	10.5, 11.5, 12.5, 13.5, 14.5^a^, 15.5	30
127	141	12.5^a^, 13.5, 14.5, 15.5^b^, 16.5, 17.5, 18.5^c^, 19.5	40
*Split dose*			
35	26	18^a^, 19^a^^,^^d^, 20, 21, 22, 23, 24, 25	40
100	66	15, 16, 17, 18, 19^e^, 20	30
120	98	12.5, 13.5, 14.5, 15.5, 16.5, 17.5	30
127	141	12, 13, 14, 15, 16^a^^,^^f^, 17^a^, 18, 19	40
Controls	−	−	10

One animal had to be excluded due to.

a mammary carcinoma (139 d, 204 d, 281 d, 249 d, 132 d, 277 d).

b bacterially induced inflammation of one hind leg (183 d).

c lipoma (291 d).

d spinal tumor (256 d).

e unknown reason (animal found dead in cage – 21 d).

f leukemia (229 d).

# other reasons (175d).

Irradiations were performed at the Heidelberg Ion Beam Therapy Center (HIT) [2] using the intensity-controlled raster scanning technique [26]. Treatment fields were optimized with the treatment planning system TRiP (Treatment planning for particles) in terms of absorbed dose [35] using a step size of 1 mm in lateral and 2 mm in longitudinal direction together with a 3 mm ripple filter. The resulting transversal profiles (Fig. 1b and c) are in good agreement with those of the previously employed carbon ion beams and remaining differences are not expected to affect the response of the rat spinal cord [36], [37]. Specified irradiation doses refer to the maximum dose at the field center at the respective spinal cord position measured with a pinpoint ionization chamber (TM31009, PTW Freiburg, Germany).

### 2.3 Follow-up and biological endpoint

Weight and general condition of the animals after irradiation were checked weekly. Once neurological disorders of the forelimbs appeared, the animals were checked more frequently. Animals were scored as responders, if they reached the biological endpoint paresis grade II (signs of paralysis at the forelimbs within 300 days after irradiation). Immediately after detection of the endpoint, the animals were sacrificed and the spinal cord was extracted for further processing.

### 2.4 Data analysis

The logistic dose–response model was adjusted to the actuarial response rates to determine the effective doses ED_50_ (dose at 50 % effect probability) for each spinal cord position in the SOBP and for both fractionation schedules [29], [30], [31], [32]. RBE-values were determined as the ratio of the ED_50_-values obtained from a previously performed reference experiment with photons [27], [28] and those of the present oxygen ion experiments. In addition, the parameters α/β and the biologically effective dose at 50 % effect probability (BED_50_) of the linear-quadratic (LQ) model [38] were adjusted by generalized logistic regression [32]. Based on the ratio of BED_50_ for photons and oxygen ions, the maximum RBE in the limit of zero fractional dose was estimated.

### 2.5 RBE model prediction

For comparison with the measured data, the RBE was calculated at the four spinal cord positions within the SOBP and for both fractionation schedules using LEM I (applied clinically for carbon ions) [9], the more recent version LEM IV [25], [39] (employing the ‘full simulation approach’ [39]) as well as the mMKM (applied clinically for carbon and recently also for helium ions [10], [40]). The RBE was calculated at the dose levels of the estimated ED_50_-values for oxygen ions of the present study. Calculations were performed with standard parameters for LEM I (α/β = 2 Gy, α = 0.1 Gy^−1^, D_t_ = 30 Gy), LEM IV (α/β = 2 Gy, α = 0.003 Gy^−1^, D_t_ = 22 Gy) [31] and mMKM (α/β = 2 Gy, α = 0.003 Gy^−1^, R_d_ = 0.3, R_n_ = 3.6 µm) [41], [42].

### 2.6 Statistics

As in the previous carbon ion studies [29], [30], [31], [32], logistic regression to the experimental response rates was performed using the maximum likelihood fitting procedure of STATISTICA [43]. Incomplete follow-up of animals was considered using the method of effective sample sizes [44] that corrects the number of treated and responding animals to match actuarial response rates and their variances. Standard errors (SE) of ED_50_, BED_50_, RBE and α/β were calculated by error propagation considering the correlation of the underlying parameters. Fieller’s theorem was used to calculate the 90 % confidence limits (CL) [45]. If the SE of ED_50_ could not be calculated by STATISTICA, it was estimated as 25 % of the dose difference between the neighboring 0 and 100 % response levels [29]. Deviations were considered as significant, if the calculated RBE was not within the 90 % CL of the experimental value.

## 3 Results

Apart from the intended biological endpoint of this study, irradiations were well tolerated by the animals. Some animals had to be excluded from the experiments due to the spontaneous development of mammary carcinoma or for other reasons (Table 1). The latency time until the onset of the endpoint paresis grade II is shown in supplementary Figure 1. Based on the slope of the linear regression, no significant dependence on LET was observed for the minimum and mean latency time (p > 0.05).

**Fig. 2:**
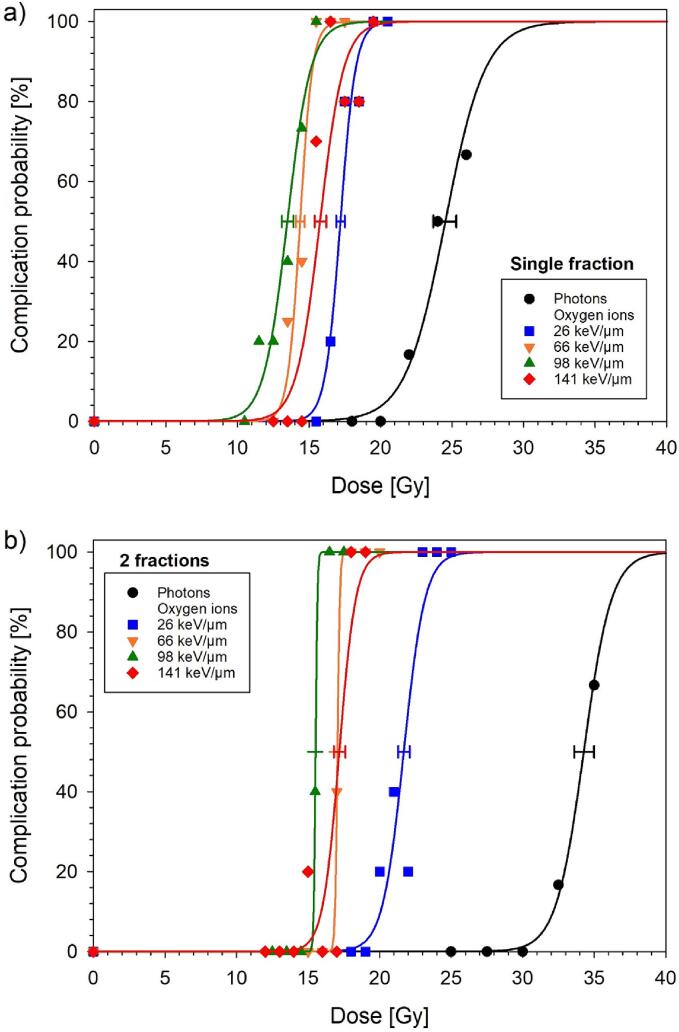
Dose-response curves for the endpoint paresis grade II after irradiation of the rat spinal cord at four different positions within the SOBP using either single (a) or split (b) doses of oxygen ions. The error bars indicate the SE of ED_50_. The reference dose–response curves for photons have been published previously [28].

**Fig. 3:**
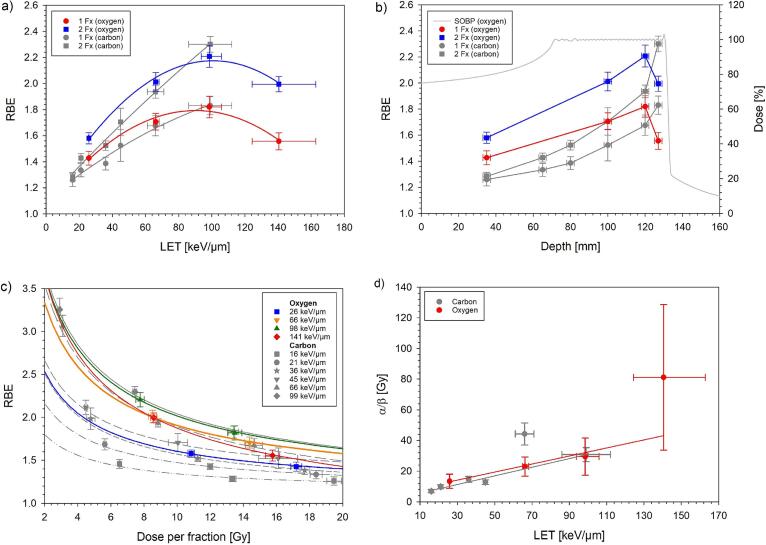
RBE of the rat spinal cord after single (1 Fx) and split (2 Fx) oxygen ion doses as a function of LET (a), depth (b) and dose per fraction (c). Additionally, the measured α/β-value is shown (d). For comparison, previously measured data for carbon ions are also displayed [29], [30], [31]. The LET-dependence of the RBE was fitted by 2nd order polynomials while the depth dependence was linearly interpolated. The dose-dependence was inter- and extrapolated based on the LQ-model using the experimentally obtained α/β-ratios. The dependence of α/β on LET was fitted using an inverse variance weighted linear regression. Horizontal error bars display the LET-uncertainty (a,d) based on a 2 mm range uncertainty (b) or the SE of ED_50_ per fraction (c), respectively, while the vertical error bars indicate the standard error of the RBE or α/β, respectively. Note: All carbon ion data are separately displayed in [32]. The large deviation of the α/β-value for carbon ions at 66 keV/µm originates from the dose–response curve for 6 fractions located at rather low doses, potentially caused by experimental uncertainties.

Fig. 2 displays the dose–response curves for single and split doses of oxygen ions at the four spinal cord positions within the SOBP. The ED_50_ values were markedly higher for split than for single doses. For both fractionation schedules, ED_50_ decreased for LET values from 26 to 98 keV/µm, but increased again at 141 keV/µm (Table 2, ED_50_ vs RBE). As a result, the RBE increased up to 1.82 (1 Fx) and 2.21 (2 Fx) at 98 keV/µm before declining again to 1.56 (1 Fx) and 1.99 (2 Fx) at the highest LET. The maximum RBE in the limit of zero fractional dose ranged between 6.23 and 12.94 (Table 2, BED_50_ vs RBE_max_). The dependence of RBE on LET, depth and dose per fraction is visualized in Fig. 3a-c. The estimated α/β-values ranged from 13.4 to 81.2 Gy (Fig. 3d, Table 2, α/β).

**Table 2:** ED_50_-, BED_50_-, RBE- and α/β-values derived from single (1 Fx) and split (2 Fx) dose irradiations of the rat spinal cord including standard errors (SE) and 90%-confidence limits (CL).

**Study**	**Measured parameters**	
***1 Fx***	***ED_50_ ± SE (90 % CL) [Gy]***	***RBE ± SE (90 % CL)***
Photons^a^	24.5 ± 0.8 (23.3 - 26.7)	−-
Oxygen ions		
26 keV/µm	17.2 ± 0.3 (16.5 - 17.9)	1.43 ± 0.05 (1.34 - 1.51)
66 keV/µm	14.4 ± 0.3 (13.7 - 15.0)	1.71 ± 0.06 (1.60 - 1.81)
98 keV/µm	13.5 ± 0.4 (12.7 - 14.4)	1.82 ± 0.08 (1.69 - 1.96)
141 keV/µm	15.8 ± 0.4 (14.9 - 16.6)	1.56 ± 0.06 (1.45 - 1.67)
***2 Fx***	***ED_50_ ± SE (90 % CL) [Gy]***	***RBE ± SE (90 % CL)***
Photons^a^	34.3 ± 0.7 (32.9 - 36.6)	−-
Oxygen ions		
26 keV/µm	21.7 ± 0.4 (20.9 - 22.4)	1.58 ± 0.04 (1.51 - 1.65)
66 keV/µm	17.0 ± 0.5 (*–*)^b^	2.01 ± 0.07 (1.90 - 2.13)
98 keV/µm	15.5 ± 0.5 (*–*)^b^	2.21 ± 0.08 (2.08 - 2.35)
141 keV/µm	17.2 ± 0.4 (16.4 - 17.9)	1.99 ± 0.06 (1.90 - 2.09)
***1 and 2 Fx***	***BED_50_ ± SE (90 % CL) [Gy]***	***RBE_max_ ± SE (90 % CL)***
Photons^c^	244.9 ± 24.3 (208.2 - 293.3)	−-
Oxygen ions		
26 keV/µm	39.3 ± 6.8 (30.7 - 59.8)	6.23 ± 1.24 (4.59 - 8.96)
66 keV/µm	23.4 ± 2.1 (20.3 - 29.1)	10.45 ± 1.39 (8.36 - 12.99)
98 keV/µm	19.7 ± 2.1 (16.7 - 25.1)	12.44 ± 1.82 (9.79 - 15.89)
141 keV/µm	18.9 ± 1.5 (16.8 - 22.4)	12.94 ± 1.63 (10.44 - 15.86)
***1 and 2 Fx***	α/β ***± SE (90 % CL) [Gy]***	
Photons^c^	2.8 ± 0.4 (2.2 - 3.5)	
Oxygen ions		
26 keV/µm	13.4 ± 4.6 (6.6 - 23.8)	
66 keV/µm	23.0 ± 6.2 (13.2 - 38.9)	
98 keV/µm	29.5 ± 12.1 (14.4 - 69.5)	
141 keV/µm	81.2 ± 47.4 (34.1 - 469.9)	

*^a^Data from*[28].

*^b^CL could not be calculated.*

*^a)^Derived in*[32]*based on data from*[28].

**Fig. 4:**
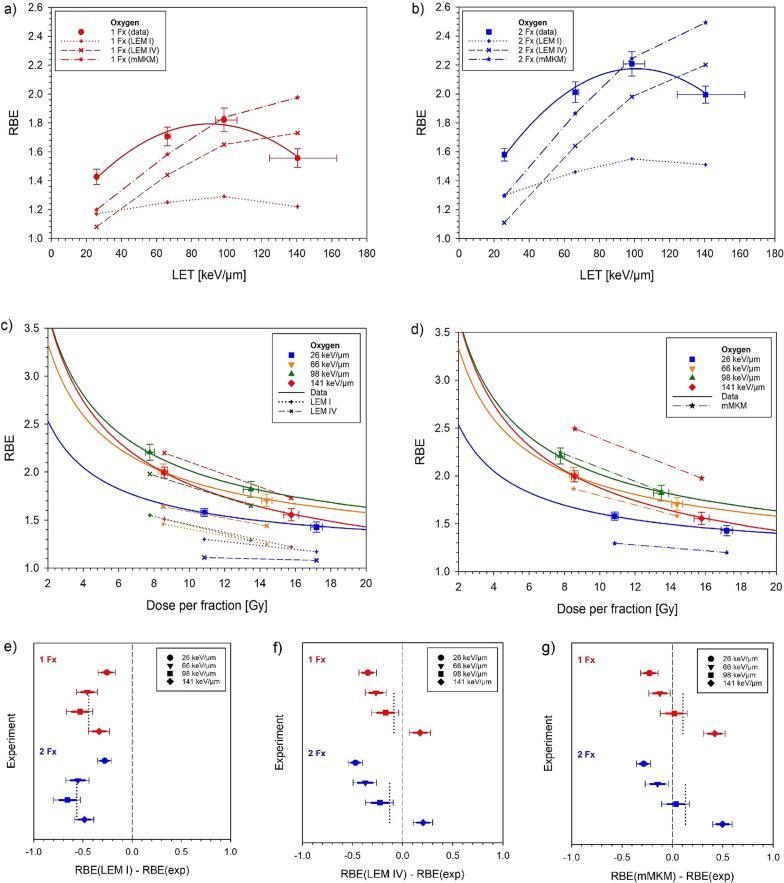
Comparison of RBE-values predicted by LEM I, LEM IV or mMKM with the measured data. RBE comparisons are displayed as a function of LET for single (a) and split (b) doses and as a function of dose per fraction for LEM I/IV (c) and mMKM (d). Deviations between predicted and measured RBE are visualized for LEM I (e), LEM IV (f) and mMKM (g). Here, horizontal error bars represent the experimental standard errors (thick line) and the 90% confidence limits (thin line) as provided in Table 2. The dotted lines indicate the average deviations in the SOBP.

Fig. 4 and supplementary Figure 2 compare measured and model-predicted RBE-values. LEM I predicted the smallest increase with LET and deviated from measurements by −0.26 (1 Fx) and −0.28 (2 Fx) in the plateau and by −0.44 ± 0.10 (1 Fx) and −0.56 ± 0.09 (2 Fx) in the SOBP (mean ± SD). In contrast, LEM IV predicted a much larger slope and the RBE deviated from measurements by −0.35 (1 Fx) and −0.47 (2 Fx) in the plateau and in average by −0.09 ± 0.23 (1 Fx) and −0.13 ± 0.30 (2 Fx) in the SOBP. Relative to LEM IV, predictions of mMKM were shifted to higher RBEs and exhibited deviations of −0.23 (1 Fx) and −0.29 (2 Fx) in the plateau and average deviations of 0.10 ± 0.28 (1 Fx) and 0.13 ± 0.33 (2 Fx) in the SOBP. None of the models predicted a significant decrease of the RBE at the highest LET.

## 4 Discussion

This study provides first *in vivo* RBE data of oxygen ions in the rat spinal cord as a function of LET and fractional dose. It thereby adds significant knowledge to the few available *in vitro* data [46]. The study used the same setup as previous experiments with protons, helium and carbon ions [29], [30], [31], [32], [33], [34]. The RBE was calculated based on ED_50_-values measured in this study and in previous photon studies. Using the same photon data for all RBE studies assures that the detected RBE differences between ion types are not affected by variations in the photon reference data, although the ED_50_ values have proven to be highly reproducible even over long periods [27], [47]. Finally, the response of the rat spinal cord has been shown to be independent of the irradiated volume as long as the irradiated segment is longer than 8 mm [36], [37].

In the present study, the RBE of oxygen ions demonstrated a strong LET- and dose-dependence. Up to 98 keV/µm, the RBE values were found to be higher than those measured previously for carbon ions at the same depth, however, since the RBE of oxygen ions in the plateau increased as well, the peak-to-plateau-ratio was slightly lower than for carbon ions at 66 and 98 keV/µm. Furthermore, the RBE at the same LET was only slightly larger (26 and 66 keV/µm) or smaller (98 keV/µm) than for carbon ions [32]. The most striking finding, however, was the unexpectedly low RBE at 141 keV/µm, which could be considered as an indication of the so-called overkill effect, i.e. a decreased effectiveness relative to photons due to extremely high local doses, although *in vitro* data for carbon ions suggest that this effect occurs only beyond 200 keV/µm [48]. While this could be a possible explanation, also experimental uncertainties have to be considered. While range uncertainties due to weight variations of the animals appear highly unlikely, it cannot be completely ruled-out that the lower RBE originates at least partly from an increased beam width relative to the day on which dosimetry was performed, although the experimental beam line at HIT is regularly checked to assure highly reproducible irradiations. With this respect, a previous study with carbon ions at 127 mm depth revealed a RBE, which was essentially the same as that at 120 mm (1.66 vs 1.68) [29], and repeating this experiment increased the RBE by 0.17 [30]. However, even when considering this increase as the potential uncertainty in the present experiment, this would still lead to a highly non-linear RBE vs LET curve for oxygen ions with comparable RBE values at 120 and 127 mm depth. Already such deviations from linearity would indicate an overkill effect, being characterized by a less pronounced effect than expected based on the energy deposition of oxygen ions. The actual decrease of RBE, however, would then only appear at very high LET values. It is noteworthy that for carbon ions, the LET-dependence has also been fitted by a 2nd order polynomial but nevertheless resulted in a strictly linear dependence for single and split doses and only in a slight bending for 6 fractions [32], comparable to that of oxygen ions found below 98 keV/µm. It has to be further noted that if the decreased RBE was actually caused by uncertainties in the beam parameter settings, it is likely that both the single and split dose irradiation would have been affected as they were performed on the same days and thus cannot be considered as completely independent. Therefore, an additional independent experiment is required to finally clarify the RBE-values beyond the LET of 98 keV/µm. If this experiment would confirm the decreased RBE, this could be of clinical advantage as the distal end of the SOBP is often located in normal tissue.

In addition to the observed LET-dependence, the RBE increased with decreasing fractional dose and extrapolation to the much lower clinically applied doses, using the LQ-model and experimentally obtained α/β-values, predicts even higher RBE-values. This extrapolation, however, has to be considered with great care due to the involved experimental uncertainties and limitations of the LQ-model when applied to large fractional doses [38].

Similar to our previous finding for carbon ions [32], the α/β-value of oxygen ions increased with rising LET and was generally much larger than for photons. This suggests a decreased repair capacity of the CNS after oxygen ion doses and thus a reduced dependence on fractionation within the SOBP region that might advocate hypofractionation.

Comparing RBE-predictions with experimental data in the range of up to 98 keV/µm revealed a stronger dependence on LET for LEM IV and mMKM, while the dependence of LEM I was much weaker. At 141 keV/µm, the predicted RBE increased further (LEM IV, mMKM) or decreased only slightly (LEM I), however, the slope decreased for all three models, which would support a trend towards a saturation of the RBE beyond 98 keV/µm. Up to this LET value, the deviations between predicted and measured RBE were smallest for the mMKM followed by LEM IV, while the deviation of LEM I was much larger. Only at the highest LET, mMKM deviated more than LEM IV. Besides the absolute deviations between predicted and measured RBEs at different LETs, the dose-dependence was well reflected by all three models, at least in the measured range.

The comparison of predicted and measured RBE data can be used to analyze the cause for the deviations, e.g. by simulations, and to refine the model parameters or the model itself. For this, *in vivo* data are especially important as the response of tissue may differ from that of cells and may therefore require additional adjustments of the model parameters. However, until such a final RBE model is available, the most suitable model has to be selected based on the available *in vitro* [49] and *in vivo* [32], [34] data. With this respect, the rat spinal cord is an ideal model system as it allows to measure the response of late reacting normal tissue to the local radiation quality of the treatment field. Similar to our previous studies with other ion types [32], [33], [34], the present study provides information on the correctness of (i) the average RBE in the target volume, and (ii) the relative dependence of the RBE on LET and dose. While deviations in average RBE lead to an over- or under-dosage of the entire target volume, they may be compensated by adapting the dose prescription accordingly. This is part of clinical dose finding studies when dealing with a radiation quality of unknown effectiveness. In contrast, deviations in the relative RBE distribution will result in local hot or cold spots within the target volume, even if the average dose is correct. Clinically, these local deviations are much more difficult to detect and experimental studies like the present one are therefore required. Finally, in spite of the slightly lower peak-to-plateau RBE ratio of oxygen, the assessment whether carbon or oxygen ions have the better therapeutic ratio has to consider also the RBE in tumors, which differs from that in normal tissue [50].

In conclusion, this study established the first RBE data for the late reacting CNS after single and split doses of oxygen ions. The data was used to validate the RBE-dependence on LET and dose in three different RBE-models. This study extends the existing data base for protons, helium and carbon ions and provides important information for the implementation of oxygen ions in patient treatments.

## 5 Sources of support

This study received funding from the German Cancer Aid (111434 and 70112975).

## CRediT authorship contribution statement

**Christin Glowa:** Conceptualization, Validation, Investigation, Writing – original draft, Visualization, Supervision. **Maria Saager:** Conceptualization, Validation, Investigation, Writing – review & editing, Supervision. **Lisa Hintz:** Validation, Investigation, Writing – review & editing. **Rosemarie Euler-Lange:** Investigation, Writing – review & editing. **Peter Peschke:** Conceptualization, Methodology, Validation, Writing – review & editing. **Stephan Brons:** Validation, Investigation, Writing – review & editing. **Michael Scholz:** Software, Formal analysis, Writing – review & editing. **Stewart Mein:** Software, Formal analysis, Writing – review & editing. **Andrea Mairani:** Software, Formal analysis, Writing – review & editing. **Christian P. Karger:** Conceptualization, Methodology, Validation, Formal analysis, Writing – original draft, Visualization, Supervision, Funding acquisition.

## Declaration of competing interest

The authors declare the following financial interests/personal relationships which may be considered as potential competing interests: Dr. Karger received funding for the present study by the German Cancer Aid (grant number 111,434 and 70112975). Dr. Scholz holds a European Patent 10 718 875.7 with royalties paid by Siemens and RaySearch. All other authors do not report any financial interests.

## References

[1] Kamada T., Tsujii H., Blakely E.A., Debus J., De Neve W., Durante M. (2015). Carbon ion radiotherapy in Japan: an assessment of 20 years of clinical experience. Lancet Oncol.

[2] Combs S.E., Jäkel O., Haberer T., Debus J. (2010). Particle therapy at the heidelberg ion therapy center (HIT) - Integrated research-driven university-hospital-based radiation oncology service in Heidelberg. Germany Radiother Oncol.

[3] Rossi S. (2015). The national centre for oncological hadrontherapy (CNAO): Status and perspectives. Phys Med.

[4] Stock M., Georg P., Mayer R., Bohlen T.T., Vatnitsky S. (2016). Development of clinical programs for carbon ion beam therapy at medaustron. Int J Part Ther.

[5] Suit H., DeLaney T., Goldberg S., Paganetti H., Clasie B., Gerweck L. (2010). Proton vs carbon ion beams in the definitive radiation treatment of cancer patients. Radiother Oncol.

[6] Karger C.P., Peschke P. (2017). RBE and related modeling in carbon-ion therapy. Phys Med Biol.

[7] Karger C.P., Glowa C., Peschke P., Kraft-Weyrather W. (2021). The RBE in ion beam radiotherapy: in vivo studies and clinical application. Z Med Phys.

[8] Kanai T., Furusawa Y., Fukutsu K., Itsukaichi H., Eguchi-Kasai K., Ohara H. (1997). Irradiation of mixed beam and design of spread-out Bragg peak for heavy-ion radiotherapy. Radiat Res.

[9] Scholz M., Kellerer A.M., Kraft-Weyrather W., Kraft G. (1997). Computation of cell survival in heavy ion beams for therapy. the model and its approximation. Radiat Environ Biophys.

[10] Inaniwa T., Furukawa T., Kase Y., Matsufuji N., Toshito T., Matsumoto Y. (2010). Treatment planning for a scanned carbon beam with a modified microdosimetric kinetic model. Phys Med Biol.

[11] Wenzl T., Wilkens J.J. (2011). Modelling of the oxygen enhancement ratio for ion beam radiation therapy. Phys Med Biol.

[12] Phillips T.L., Fu K.K., Curtis S.B. (1977). Tumor biology of helium and heavy ions. Int J Radiat Oncol Biol Phys.

[13] Tenforde T.S., Tenforde S.D., Crabtree K.E., Parks D.L., Schilling W.A., Parr S.S. (1981). RBE values for radiation-induced growth delay in rat rhabdomyosarcoma tumors exposed to plateau and peak carbon, neon and argon ions. Int J Radiat Oncol Biol Phys.

[14] Goldstein L.S., Phillips T.L., Fu K.K., Ross G.Y., Kane L.J. (1981). Biological effects of accelerated heavy ions. I. Single doses in normal tissue, tumors, and cells in vitro. Radiat Res.

[15] Tenforde T.S., Curtis S.B., Crabtree K.E., Tenforde S.D., Schilling W.A., Howard J. (1980). In vivo cell survival and volume response characteristics of rat rhabdomyosarcoma tumors irradiated in the extended peak region of carbon- and neon-ion beams. Radiat Res.

[16] Wheeler K.T., Deen D.F., Leith J.T., Norton K.L. (1979). Cellular response of a rat brain tumor to a therapeutic carbon ion beam. Radiology.

[17] Leith J.T., Lewinsky B.S., Woodruff K.H., Schilling W.A., Lyman J.T. (1975). Tolerance of the spinal cord of rats to irradiation with cyclotron-accelerated helium ions. Cancer.

[18] Leith J.T., Woodruff K.H., Lewinsky B.S., Lyman J.T., Tobias C.A. (1975). Letter: Tolerance of the spinal cord of rats to irradiation with neon ions. Int J Radiat Biol Relat Stud Phys Chem Med.

[19] Leith J.T., Woodruff K.H., Howard J., Lyman J.T., Smith P., Lewinsky B.S. (1977). Early and late effects of accelerated charged particles on normal tissues. Int J Radiat Oncol Biol Phys.

[20] Leith J.T., McDonald M., Powers-Risius P., Bliven S.F., Howard J. (1982). Response of rat spinal cord to single and fractionated doses of accelerated heavy ions. Radiat Res.

[21] Rodriguez A, Alpen EL, DeGutzman R, Pioleau J. Irradiation of rat thoraco-lumbar spinal cord with fractionated doses of helium and neon ions. Proceedings of the 8th International Congress of Radiation Research. 1987;1:249.

[22] Rodriguez A, Levy RP, Fabrikant JI. Experimental central nervous system injury after charged-particle irradiation. In: Gutin PH, Leibel SA, Sheline GE, editors. Radiation injury to the nervous system: Raven Press, New York; 1991. p. 149-82.

[23] Okada S., Okeda R., Matsushita S., Kawano A. (1998). Histopathological and morphometric study of the late effects of heavy-ion irradiation on the spinal cord of the rat. Radiat Res.

[24] Kanai T., Endo M., Minohara S., Miyahara N., Koyama-ito H., Tomura H. (1999). Biophysical characteristics of HIMAC clinical irradiation system for heavy-ion radiation therapy. Int J Radiat Oncol Biol Phys.

[25] Elsässer T., Weyrather W.K., Friedrich T., Durante M., Iancu G., Krämer M. (2010). Quantification of the relative biological effectiveness for ion beam radiotherapy: direct experimental comparison of proton and carbon ion beams and a novel approach for treatment planning. Int J Radiat Oncol Biol Phys.

[26] Haberer T., Becher W., Schardt D., Kraft G. (1993). Magnetic scanning system for heavy ion therapy. Nucl Instrum Meth.

[27] Debus J., Scholz M., Haberer T., Peschke P., Jakel O., Karger C.P. (2003). Radiation tolerance of the rat spinal cord after single and split doses of photons and carbon ions. Radiat Res.

[28] Karger C.P., Peschke P., Sanchez-Brandelik R., Scholz M., Debus J. (2006). Radiation tolerance of the rat spinal cord after 6 and 18 fractions of photons and carbon ions: experimental results and clinical implications. Int J Radiat Oncol Biol Phys.

[29] Saager M., Glowa C., Peschke P., Brons S., Scholz M., Huber P.E. (2014). Carbon ion irradiation of the rat spinal cord: dependence of the relative biological effectiveness on linear energy transfer. Int J Radiat Oncol Biol Phys.

[30] Saager M., Glowa C., Peschke P., Brons S., Grün R., Scholz M. (2016). The relative biological effectiveness of carbon ion irradiations of the rat spinal cord increases linearly with LET up to 99 keV/µm. Acta Oncol.

[31] Saager M., Glowa C., Peschke P., Brons S., Grün R., Scholz M. (2015). Split dose carbon ion irradiation of the rat spinal cord: dependence of the relative biological effectiveness on dose and linear energy transfer. Radiother Oncol.

[32] Saager M., Glowa C., Peschke P., Brons S., Grun R., Scholz M. (2020). Fractionated carbon ion irradiations of the rat spinal cord: comparison of the relative biological effectiveness with predictions of the local effect model. Radiat Oncol.

[33] Saager M., Peschke P., Brons S., Debus J., Karger C.P. (2018). Determination of the proton RBE in the rat spinal cord: Is there an increase towards the end of the spread-out Bragg peak?. Radiother Oncol.

[34] Hintz L., Glowa C., Saager M., Euler-Lange R., Peschke P., Brons S. (2022). Relative biological effectiveness of single and split helium ion doses in the rat spinal cord increases strongly with linear energy transfer. Radiother Oncol.

[35] Krämer M., Jäkel O., Haberer T., Kraft G., Schardt D., Weber U. (2000). Treatment planning for heavy-ion radiotherapy: physical beam model and dose optimization. Phys Med Biol.

[36] Hopewell J.W., Morris A.D., Dixon-Brown A. (1987). The influence of field size on the late tolerance of the rat spinal cord to single doses of X rays. Br J Radiol.

[37] van der Kogel A.J. (1993). Dose-volume effects in the spinal cord. Radiother Oncol.

[38] Fowler J.F. (1989). The linear-quadratic formula and progress in fractionated radiotherapy. Br J Radiol.

[39] Friedrich T., Scholz U., Elsässer T., Durante M., Scholz M. (2012). Calculation of the biological effects of ion beams based on the microscopic spatial damage distribution pattern. Int J Radiat Biol.

[40] Tessonnier T., Ecker S., Besuglow J., Naumann J., Mein S., Longarino F.K. (2023). Commissioning of helium ion therapy and the first patient treatment with active beam delivery. Int J Radiat Oncol Biol Phys.

[41] Magro G., Dahle T.J., Molinelli S., Ciocca M., Fossati P., Ferrari A. (2017). The FLUKA Monte Carlo code coupled with the NIRS approach for clinical dose calculations in carbon ion therapy. Phys Med Biol.

[42] Mairani A., Magro G., Tessonnier T., Bohlen T.T., Molinelli S., Ferrari A. (2017). Optimizing the modified microdosimetric kinetic model input parameters for proton and (4)He ion beam therapy application. Phys Med Biol.

[43] StatSoft Inc. STATISTICA für Windows [Software-System für Datenanalyse] . www.statsoft.com. 2011.

[44] Walker A.M., Suit H.D. (1983). Assessment of local tumor control using censored tumor response data. Int J Radiat Oncol Biol Phys.

[45] Finney D.J. (1978).

[46] Dokic I., Mairani A., Niklas M., Zimmermann F., Chaudhri N., Krunic D. (2016). Next generation multi-scale biophysical characterization of high precision cancer particle radiotherapy using clinical proton, helium-, carbon- and oxygen ion beams. Oncotarget.

[47] Saager M., Hahn E.W., Peschke P., Brons S., Huber P.E., Debus J. (2020). Ramipril reduces incidence and prolongates latency time of radiation-induced rat myelopathy after photon and carbon ion irradiation. J Radiat Res.

[48] Weyrather W.K., Ritter S., Scholz M., Kraft G. (1999). RBE for carbon track-segment irradiation in cell lines of differing repair capacity. Int J Radiat Biol.

[49] Monini C., Alphonse G., Rodriguez-Lafrasse C., Testa E., Beuve M. (2019). Comparison of biophysical models with experimental data for three cell lines in response to irradiation with monoenergetic ions. Phys Imaging Radiat Oncol.

[50] Glowa C., Peschke P., Brons S., Debus J., Karger C.P. (2021). Effectiveness of fractionated carbon ion treatments in three rat prostate tumors differing in growth rate, differentiation and hypoxia. Radiother Oncol.

