# Supplementary material for: Relative biological effectiveness of oxygen ion beams in the rat spinal cord: Dependence on linear energy transfer and dose and comparison with model predictions
Source: Phys Imaging Radiat Oncol. 2024 Apr 20;30:100581. doi: 10.1016/j.phro.2024.100581 (PMC11070926; doi:10.1016/j.phro.2024.100581)
Supplement: Supplementary Data 1 [file mmc1.pdf]

Supplementary material

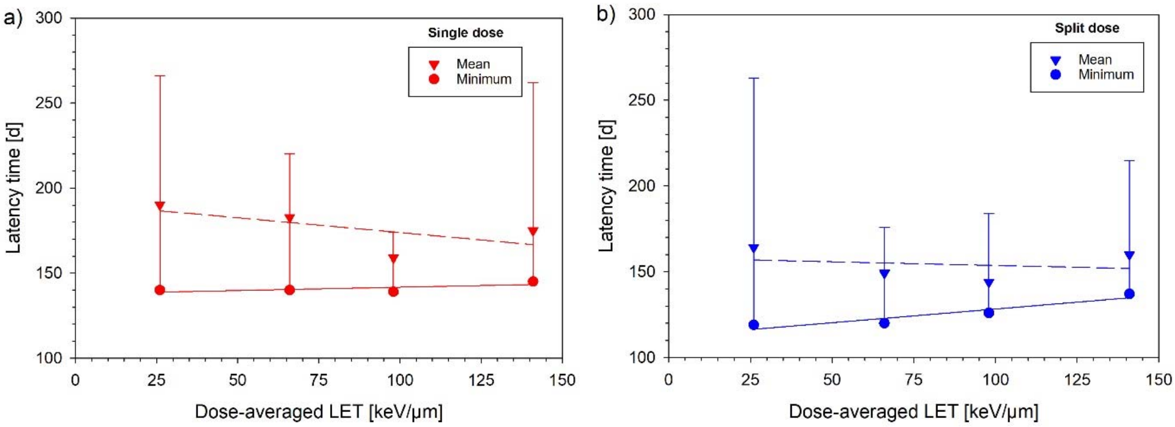

Supplementary Figure 1. Minimum and mean latency time until onset of the endpoint paresis grade II as a function of LET after single (a) and split (b) doses of oxygen ions. The error bars indicate the range of latency times for each experiment.

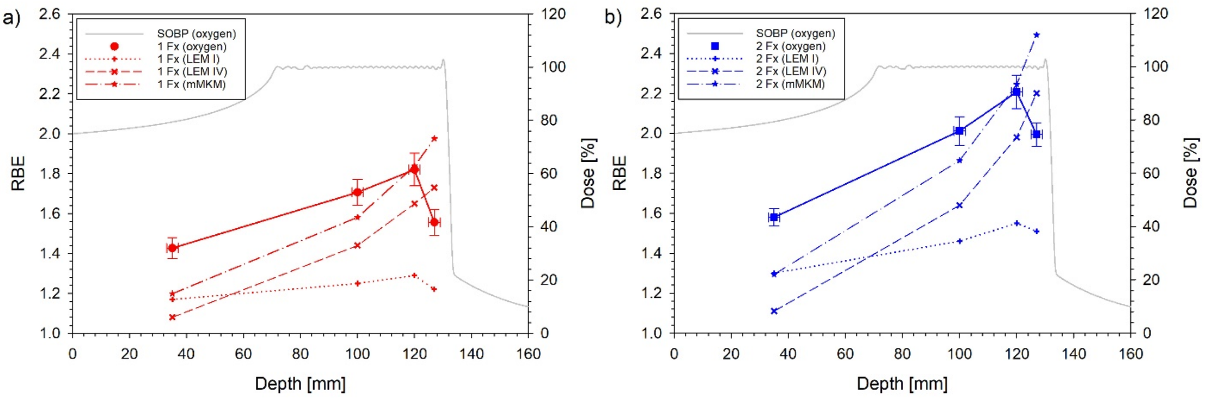

Supplementary Figure 2. Comparison of RBE-values predicted by LEM I, LEM IV or mMKM with the measured data as a function of depth for single (a) and split (b) doses.
